# Assessment of the feasibility of including community pharmacies under the regulation of the saudi food and drug authority

**DOI:** 10.1007/s44446-025-00036-0

**Published:** 2025-10-02

**Authors:** Ahmed H. Alanazi, Hessa H. Alhaidar, Mohammad A. Altamimi

**Affiliations:** Department of Pharmaceutics, College of Pharmacy, King Saud University, Riyadh, 11451 Saudi Arabia

**Keywords:** Saudi food and drug authority, Ministry of health, Community pharmacies, Cross-sectional study

## Abstract

**Abstract:**

In Saudi Arabia, the regulation of community pharmacies currently falls under the Ministry of Health (MOH). There is a need to shift the regulatory framework of community pharmacies to be under the Saudi Food and Drug Authority (SFDA) to align with global standards. However, there is limited knowledge about the perceptions of pharmacists regarding the current regulatory framework in community pharmacies in the Kingdom of Saudi Arabia (KSA).

**Objectives:**

This study aims to assess pharmacists' knowledge, perceptions, and preferences regarding the current regulatory framework of community pharmacies and feasibility of transitioning regulatory oversight to SFDA.

**Methodology:**

A cross-sectional survey design was used to assess the regulation of community pharmacies in Saudi Arabia. The sample consisted of 139 pharmacists from various sectors, selected through random sampling. A structured questionnaire focusing on regulation, awareness, and perceptions was distributed online. The questionnaire's validity and reliability were ensured through expert review. Descriptive statistics in SPSS were used for data analysis.

**Results:**

The survey findings revealed a diverse representation across the pharmaceutical sector. Hospital pharmacists formed the largest group (37.4%, n = 52), followed by regulatory field workers from the Ministry of Health (21.6%, n = 30) and SFDA (20.1%, n = 28), pharmaceutical industry professionals (11.5%, n = 16), and community pharmacists (9.4%, n = 13). Most participants (51.8%, n = 72) had 1–5 years of experience, while 31.7% (n = 44) had 6–10 years, and 16.5% (n = 23) had more than 10 years of experience. Regarding current regulatory oversight, the majority (82.7%, n = 115) reported being under MOH regulation, with 12.2% (n = 17) under SFDA oversight. The study revealed high awareness of current regulations (77.0%, n = 107), though most participants (62.6%, n = 87) expressed dissatisfaction with the current regulatory framework. Notably, 77.0% (n = 107) preferred SFDA as the future regulator, and 80.6% (n = 112) believed SFDA would perform better in regulating the sector. Most participants demonstrated strong agreement with proposed regulatory changes, with 78.4% (n = 109) agreeing to P1 and 79.1% (n = 110) to P4 statements regarding regulatory reform.

**Conclusion:**

The study reveals strong sector-wide preference among pharmaceutical professionals for transitioning community pharmacy regulation to SFDA, driven by dissatisfaction with current MOH oversight. This consensus across all professional categories and experience levels indicates practitioners' expectations that SFDA regulation would enhance pharmaceutical service quality and regulatory effectiveness in Saudi Arabia.

**Supplementary Information:**

The online version contains supplementary material available at 10.1007/s44446-025-00036-0.

## Introduction

The Saudi Food and Drug Authority (SFDA), established in 2003 as an independent regulatory body with its own board, is the government agency responsible for regulating domestic and imported food, drugs, cosmetics and medical devices. However, community pharmacies—retail establishments that primarily engage in dispensing medications and providing healthcare advice to the public—remain under the exclusive oversight of the Ministry of Health (MOH), the government agency responsible for healthcare policy development and implementation. The integration of community pharmacies into a more centralized regulatory framework would ensure better regulation of medication dispensing and counseling, contributing to the overall improvement of the healthcare system in the country (Al-Jedai et al. 2016; Al-Mohamadi et al. 2013). The status of pharmaceutical care in Saudi community pharmacies, as well as the varying practices across the nation, highlight the need for standardized regulations (Alfadl et al. 2018; Alghadeer and Al-Arifi 2021). The adaptability and preparedness of community pharmacies during crises, such as the COVID-19 pandemic, further emphasize the importance of effective pharmaceutical governance (Khojah 2020).

Alghaith et al., have reported that the inclusion of community pharmacies under the less fragmented oversight would strengthen the pharmaceutical system in the kingdom (Alghaith et al. 2020). Jairoun et al., also address the use of herbal supplements and the need for improved surveillance in their dispensing, providing more stringent regulation in this area (Jairoun et al. 2022). The current status of pharmaceutical care in Saudi community pharmacies and possibilities for improvement are also vital (Alanazi et al. 2016). The role of community pharmacists in regulating public access to prescription drugs, as discussed by Bahnassi, could benefit from structured regulatory practices (Bahnassi 2016).

The significance of this research lies in its potential to provide comprehensive insights into the current state and future direction of community pharmacy regulation in Saudi Arabia. The study aims to explore the perspectives of professionals across the pharmaceutical sectors, shedding light on the perceived effectiveness and limitations of the existing regulatory framework overseen by the Ministry of Health. Additionally, the study will examine the potential benefits of transitioning to regulation by the Saudi Food and Drug Authority (SFDA). Understanding these perspectives is crucial for policymakers and stakeholders, as it will guide informed decision-making to enhance the quality and safety of healthcare services provided by community pharmacies. The study's findings are expected to identify areas where the current regulatory system may fall short and highlight improvements that could result from a more specialized regulatory approach.

Furthermore, the research is significant for its potential to address the gaps in awareness about current regulations among pharmaceutical professionals. This aspect is critical for ensuring compliance and enhancing the standard of pharmaceutical practices. By identifying these gaps and understanding their root causes, the study can inform targeted educational and training programs, improving the overall standard of pharmaceutical care. Additionally, the research will explore the challenges faced by community pharmacies under the current regulatory system and the expected impact of SFDA regulation.

## Research literature

### Literature review

Boyle et al., provides critical insights into the role of pharmacy regulatory authorities in quality improvement systems. Their study of Canadian pharmacy regulators identified five essential domains for effective pharmacy regulation: articulating compliance requirements, managing regulatory role conflicts, educational initiatives to strengthen reporting mechanisms, the advancement of quality improvement processes, and the application of coherent regulatory standards. The study revealed that regulatory bodies must carefully balance their educational and enforcement roles, suggesting that specialized agencies may be better positioned to manage these competing demands. This finding has particular relevance for Saudi Arabia's proposed regulatory transition, as it highlights the importance of establishing clear compliance markers while allowing pharmacies sufficient autonomy to implement standards appropriately within their specific contexts (Boyle et al. 2014). Fung et al., examinines disciplinary actions in pharmacy practice and demonstrates the benefits of structured regulatory environments. Their research revealed significantly lower rates of disciplinary actions among hospital pharmacists compared to community pharmacists, attributing this difference to more robust organizational frameworks and support systems. The study identified several protective factors in hospital settings that could inform community pharmacy regulation: comprehensive organizational discipline processes, practice environments promoting competence, professional representation, and emphasis on remedial approaches to practice issues (Fung et al. 2022).

Community pharmacies play a vital role in the healthcare systems of many countries, often serving as the first point of contact for patients seeking care. A review of the literature reveals that the formation, regulation, and practices of community pharmacies vary widely across different Asian countries (Aljohani, et al. 2023). The number of pharmacies has grown rapidly in recent years. However, studies suggest that the quality of care provided by these pharmacies is often limited by factors such as inadequate staff training, profit-driven practices, and weak regulatory oversight (Aljohani, et al. 2023). Also, common challenges emerge across the different regions, including the sale of prescription medications without a prescription, limited patient counseling, and poor adherence to best practices for medication storage and handling. This research offers valuable insights for Saudi Arabi’s proposed transition to more specialized regulatory authority (Aljohani, et al. 2023).

Research examining the implementation of quality frameworks in community pharmacy settings emphasizes the critical role of integration within broader healthcare systems. Hindi et al., comprehensive review highlights that successful pharmacy service delivery requires careful attention to interprofessional collaboration and effective information sharing between community pharmacies and other primary care providers. Their analysis revealed that integration encompasses both operational aspects, such as shared communication systems and clearly defined collaboration mechanisms, and professional relationships built on mutual understanding of roles and responsibilities. The study identified specific implementation requirements including established protocols for interprofessional communication, systems for sharing patient information securely, and clear documentation processes for pharmacy interventions. These findings have direct relevance for Saudi Arabia's proposed regulatory transition, suggesting that new oversight frameworks should explicitly address integration mechanisms and support the development of collaborative relationships between community pharmacies and other healthcare providers. The evidence indicates that regulatory bodies play a crucial role in establishing and maintaining these integration systems, particularly through policies that facilitate information sharing while protecting patient confidentiality and promoting interprofessional collaboration (Hindi et al. 2024).

### Regulatory frameworks in the Middle East and North Africa (MENA)

The MENA region demonstrates diverse approaches to pharmaceutical regulation, reflecting different governance structures and healthcare priorities. In Saudi Arabia, the pharmaceutical establishment system encompasses various facilities involved in pharmaceutical and herbal preparations, including factories, warehouses, scientific offices, and consultation centers. While community pharmacies are crucial healthcare access points, they are notably excluded from the System of Pharmaceutical Facilities, Pharmaceuticals and Herbal Preparations established in 2020, remaining under separate regulatory oversight (Saudi Food and Drug Authority 2020; Main Ministry of Health Office 2020).

The United Arab Emirates (UAE) employs a more distributed regulatory model, where authority is shared between the Ministry of Health, Health Authority Abu Dhabi (HAAD), and Dubai Health Authority (DHA). This multi-tiered approach allows for more localized oversight while maintaining national standards. Community pharmacies in Dubai, for instance, are subject to dual oversight from both DHA and the Ministry of Health, while other emirates fall under the Ministry's purview. This system demonstrates how specialized regulatory bodies can complement traditional ministerial oversight (Main Ministry of Health Office 2019; Dubai Health Authority 2021; Main Ministry of Health 2015).

Qatar's regulatory framework categorizes pharmaceutical establishments into distinct functional groups, including commercial pharmacies, government pharmacies, private pharmacies, and pharmaceutical stores. The Health Facilities Licensing and Accreditation Department sets and enforces national standards, ensuring compliance with international best practices. This structured approach facilitates clear delineation of responsibilities and standards across different pharmacy categories (Main Ministry of Health Office 1983; Main Ministry of Health Office 2019).

Jordan's model presents an integrated approach through its Food and Drug Administration, which oversees all aspects of pharmaceutical activity from manufacturing to dispensing. The Department of Control and Inspection within the FDA monitors both locally produced and imported medicines, ensuring compliance with good manufacturing practices. This centralized oversight through a specialized agency offers potential lessons for regulatory reform in other MENA countries (Main Ministry of Health Office 2013).

Egypt's system centers on the Egyptian Drug Authority, which comprehensively regulates pharmaceutical activities, including manufacturing compliance, institutional oversight, and supply chain management. This authority's broad mandate extends to enforcing standards and conducting compliance checks, demonstrating the potential effectiveness of a specialized regulatory body (Egyptian Medicine Authority 2015).

In Iraq’s system, a pharmacy is defined as a place where prescriptions, medicines, chemicals, and other preparations are prepared and dispensed (Presidency of the Republic of Iraq 1970). Various entities, including the Inspection Department of the Ministry of Health and the Pharmacists Syndicate, have authority to inspect pharmacies and stores dealing with medicines. The Inspection Department oversees a wide range of medical professions and shops, including pharmacies and clinics (Main Ministry of Health Office 1981). The Pharmacists Syndicate appoints inspectors to monitor compliance with the Pharmacists Syndicate Law and to inspect pharmacies, apothecaries, and dealers in natural plants (Iraqi Pharmacists Syndicate 1976).

### International regulatory models

The United States presents a robust regulatory framework where community pharmacies operate under dual oversight from the Food and Drug Administration (FDA) and the National Association of Boards of Pharmacy (NABP). This system emphasizes public protection through strict record-keeping requirements, patient education mandates, and adverse event reporting protocols (National Association of Boards of Pharmacy 2024). California's approach particularly highlights the prioritization of public safety in pharmacy regulation, demonstrating how regional authorities can enhance national standards (California State Board of Pharmacy 2023).

India employs a multi-tiered regulatory system that balances national standards with state-level implementation. The Pharmacy Council of India (PCI) establishes national standards for pharmacy education and practice, while State Pharmacy Councils handle implementation at the local level (Pharmacy Council of India (PCI) n.d.). This cooperative approach between central and state authorities offers insights into managing pharmacy regulation in geographically diverse nations. The Central Drugs Standard Control Organization (CDSCO) provides additional oversight for drug manufacturing and distribution, creating clear separation between drug regulation and pharmacy practice governance (Central Drugs Standard Control Organization 2022).

China's regulatory framework, overseen by the National Medical Products Administration (NMPA), emphasizes coordination between national and provincial authorities. This system demonstrates how hierarchical regulatory structures can maintain consistent standards while accommodating regional variations (National Medical Products Administration n.d.; China CFDA(sfda) Approval n.d.). The CFDA's role in ensuring compliance with national and regional standards highlights the importance of coordinated oversight in large, diverse countries.

Japan's regulatory system operates through the Ministry of Health, Labour and Welfare (MHLW) in conjunction with the Pharmaceuticals and Medical Devices Agency (PMDA). This dual-agency approach combines broad healthcare policy oversight with specialized pharmaceutical regulation (Pharmaceuticals and Medical Devices Agency 2023; Ministry of Health, Labour and Welfare n.d.). The PMDA's independent administrative status allows for focused attention on pharmaceutical safety and quality while maintaining coordination with broader healthcare governance.

The European Union presents a unique model where overarching pharmaceutical regulations coexist with member state autonomy in community pharmacy oversight. The EU's framework, particularly Directive 2001/83/EC, establishes common standards while allowing national authorities to implement context-appropriate regulations (World Health Organization 2019; European Parliament and Council of the European Union n.d.). This balance between harmonization and local adaptation offers valuable insights for countries seeking to modernize their regulatory frameworks.

### Implications for Saudi Arabia

This international review reveals several key considerations for Saudi Arabia's regulatory reform efforts. First, the trend toward specialized regulatory bodies for pharmaceutical oversight, as seen in Jordan and Egypt, suggests potential benefits in transitioning community pharmacy regulation to the SFDA. Second, the success of multi-tiered systems in the UAE and India demonstrates how divided regulatory responsibilities can enhance rather than impede effective oversight when properly coordinated.

The various models also highlight the importance of clear delineation of responsibilities between regulatory bodies, robust enforcement mechanisms, and systems for stakeholder engagement. Advanced regulatory frameworks consistently emphasize public safety, professional standards, and systematic quality assurance, elements that could inform Saudi Arabia's regulatory evolution.

As Saudi Arabia considers regulatory reform, these international examples provide valuable insights into potential structures and implementation strategies. The diversity of successful approaches suggests that effective regulation can be achieved through various models, provided they are appropriately adapted to local contexts and supported by robust implementation mechanisms.

## Methodology and procedures

### Research design

This study employed a cross-sectional survey to assess the perceptions of pharmacists from the regulatory sector, pharmaceutical firms, and manufacturing organizations regarding the feasibility of including community pharmacies under the Saudi Food and Drug Authority's (SFDA) regulation. The design provided the current attitudes and opinions among experts in the pharmaceutical field, allowing for a comprehensive understanding of the prevailing views on the potential transition.

### Research population

The research population for this study consisted of pharmacists working in various sectors of Saudi Arabia's pharmaceutical field, including the regulatory sector in SFDA and MOH, pharmaceutical firms, Community pharmacists and hospital Pharmacists.

### Sample size and sampling strategy

To determine the feasibility of including community pharmacies under the Saudi Food and Drug Authority's regulation, a statistical power analysis tool (G*Power 3.1.9.6) was used with the gathered sample data. The calculation for chi square test family, was based on a medium effect size of 0.31, an alpha level of 0.05, at 80% power. These parameters were selected to ensure that the study would have sufficient power to detect a medium-sized effect, which is usually appropriate for social science research. The power analysis resulted in a final sample size of 134 (response collected for this study is 139), which was considered adequate for producing reliable and valid results while also being feasible in terms of study resources and time. The sampling strategy implemented in this study was random sampling. This approach was chosen because it is effective in ensuring that the sample is representative and that potential biases are minimized. In using this strategy, every eligible pharmacist in the pharmaceutical field in Saudi Arabia, including those working in government sectors, companies, and manufacturers, had an equal chance of being selected.

### Inclusion and exclusion criteria

#### Inclusion criteria

The study included licensed pharmacists working in Saudi Arabia with at least one year of professional experience in the pharmaceutical sector. All participants were required to voluntarily agree to take part in the study and provide informed consent, acknowledging their understanding of the study's purpose and their role in it.

#### Exclusion Criteria

Participants not meeting one or more of the previous inclusion criteria were excluded from this study.

### Data collection instrument

The study used a structured questionnaire that was developed by the researchers, which was based on existing regulations governing community pharmacies, as enforced by governmental authorities. The questionnaire, in its primary form, comprised the following sections:

The first section of the questionnaire focused on Demographics and Professional Background. It included two items: the first item aimed to identify the participant's current position among various roles such as Hospital Pharmacist (Government/Private Sector), Regulatory Fields (Ministry of Health/SFDA), and others. The second item assessed participants'years of professional experience in the pharmaceutical sector, categorized into three groups: 1–5 years, 6–10 years, and more than 10 years of experience.

The second section, Regulation and Awareness, consisted of two items as well. The first item asked participants to identify the current regulatory body for community pharmacies, with choices like the SFDA or Ministry of Health. The second item was a Yes/No question related to the participant's awareness of current regulations governing community pharmacies.

Section three explored Perceptions of Current Regulations, with two items. The first item aimed to determine whether participants believed existing regulations effectively resolve challenges faced by community pharmacies. The second item focused on their perception of the quality of healthcare services provided by community pharmacies.

The fourth section, Preference for Regulatory Body, contained two items. The first item sought participants'preference between the SFDA and the Ministry of Health as the regulatory body for community pharmacies. The second item asked whether participants believed that regulation under the SFDA would improve healthcare services, requiring a binary response.

Finally, the fifth section, Perceptions towards SFDA Regulation, was the most extensive with five items. Each item followed a Likert-type format, allowing participants to choose from options such as Agree, Neutral, and Disagree. This section covered various aspects, including the enhancement of safety and quality standards, improvement in oversight of pharmaceutical practices, and potential burdens or resistance from industry stakeholders under SFDA regulation.

### Validity and reliability

#### Validity

Content validity of the questionnaire was carefully performed by involving a panel of 6 experts in the field of pharmacy. This panel of experts include teaching staff at pharmacy colleges, managers, and hospital pharmacists, all of whom possessed extensive knowledge and experience in the pharmaceutical sector. The experts were invited to review the questionnaire and provide their expert opinions and comments on the appropriateness, relevance, and clarity of the questionnaire items.

The purpose of this expert review process was to ensure that the questionnaire thoroughly covered all the necessary aspects related to the study's focus. Additionally, it aimed to ensure that each item was clearly and appropriately phrased to elicit precise responses from the participants. The feedback received from these experts was thoughtfully considered, and necessary modifications were made to the questionnaire to enhance its content validity. These modifications were made with the aim of accurately reflecting the research objectives and ensuring that the questionnaire was easily understandable for the target respondents.

#### Reliability

To assess the reliability of the questionnaire, the Perception responses from the participants were analyzed using Cronbach's alpha coefficient. This internal consistency measure was found to be ~ 0.66, indicating a debatable level of internal consistency among the questionnaire items.

### Data collection process

The data collection process for this study was meticulously planned and executed to ensure a comprehensive and efficient gathering of information from the target participants. The primary tool used for data collection was a validated questionnaire, which was uploaded to Google Forms, an online platform that enables easy distribution and collection of survey responses. This digital format was chosen for its accessibility, user-friendliness, and efficient data compilation capabilities. The questionnaire link was shared through various social media channels, specifically those popular and frequently used by professionals in the pharmaceutical sector in Saudi Arabia. This approach ensured a wider reach within the pharmaceutical field.

The questionnaire was made available online for two weeks, providing sufficient time for participants to notice, access, and complete the survey while keeping the data collection phase concise and efficient. To encourage participation and improve the response rate, gentle reminders were sent out through the same social media channels used for the initial distribution. These reminders were designed to motivate individuals who may have missed or overlooked the initial invitation to participate in the study. After the two-week period, the questionnaire was closed to new responses, marking the conclusion of the data collection phase. Google Forms automatically collected and organized the responses, simplifying the subsequent data analysis processes.

### Data analysis

The data analysis plan for this study was designed to meticulously examine the responses collected through the questionnaire. Initially, the data underwent a thorough organizing process to ensure accuracy and completeness. This preparatory step was crucial in identifying and rectifying any inconsistencies or missing data. Subsequently, these data were analyzed using the Statistical Analysis System (SAS, version 9.4; SAS Institute Inc., Cary, NC, USA), a comprehensive tool chosen for its robust statistical capabilities and efficiency. The analysis predominantly focused on descriptive statistics, employing frequencies and percentages to succinctly summarize and interpret the participant's responses to the questionnaire items. Furthermore, Chi-square and Cramer’s V tests were performed to examine relationships between different variables and only A significance level of p < 0.05 was specified as significant.

## Results

### Demographic characteristics of participants

**Fig. 1:**
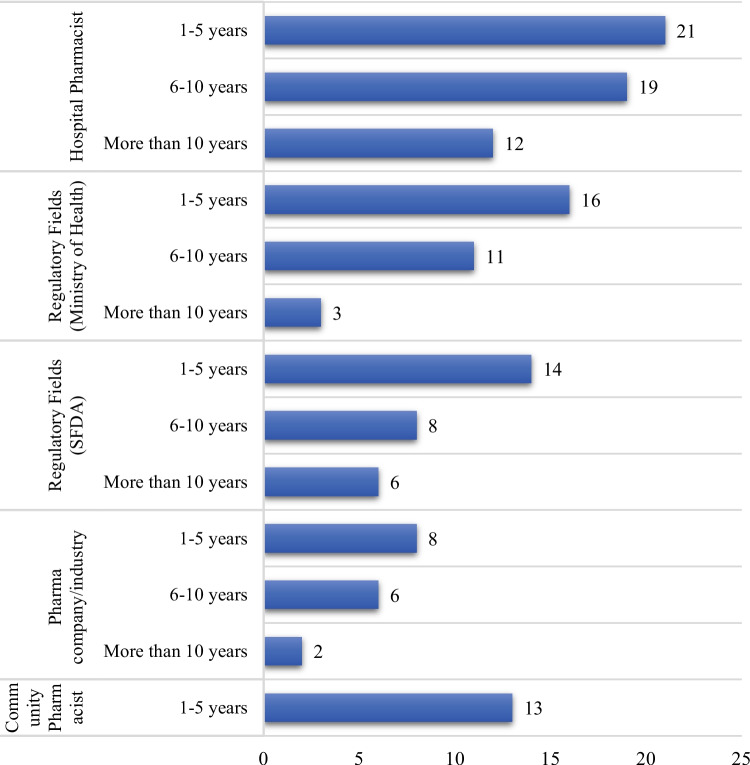
Participants' distribution based on their profession/occupation and years of experience

In this study, a total of 139 respondents have participated, see Fig. 1. The largest group in this study was hospital pharmacists in the government sector. Out of 139 respondents, 52 (or 37.4%) were hospital pharmacists in the government sector. The next largest groups were individuals working in regulatory fields associated with the Ministry of Health and the Saudi Food and Drug Authority (SFDA) at 30 (or 21.6%) and 28 (or 20.1%) participants, respectively. Pharma Company and Community pharmacists accounted for 16 (or 11.5%) and 13 (or 9.4%) of the participants, respectively.

The professional experience distribution of participants, as illustrated in Fig. 1, reveals a predominantly young workforce. Most participants 72 individuals (51.8%) have 1–5 years of experience, indicating a significant entry-level and early-career segment. The next largest group comprises 44 participants (31.7%) with 6–10 years of professional experience, suggesting a growing mid-career professional population. Only 16.5% of participants have over 10 years of experience, which points to a sector characterized by relatively recent professional entrants and ongoing workforce development.

**Fig. 2:**
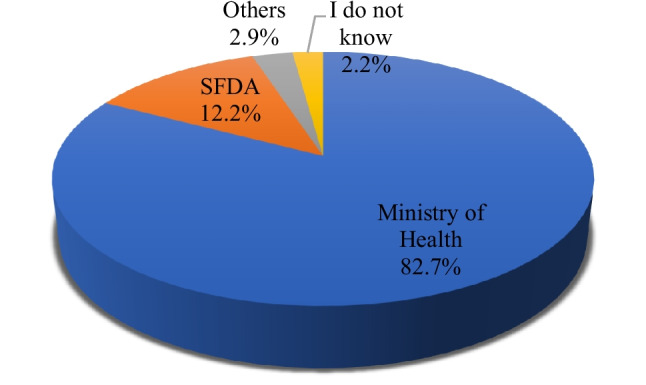
Current knowledge of community pharmacies regulatory body

In Fig. 2, (82.7%) of respondents reported that community pharmacies in Saudi Arabia are currently regulated by the Ministry of Health. A smaller percentage (12.2%) indicated that the regulatory body is the Saudi Food and Drug Authority (SFDA). Additionally, the remaining 5.0% of respondents were divided between those who identified alternative regulatory entities beyond MOH and SFDA (2.9%) and those who responded,"I do not know"(2.2%). These insights provide an understanding of the current regulatory landscape in relation to community pharmacies in Saudi Arabia.

### Current regulatory framework assessment

**Fig. 3:**
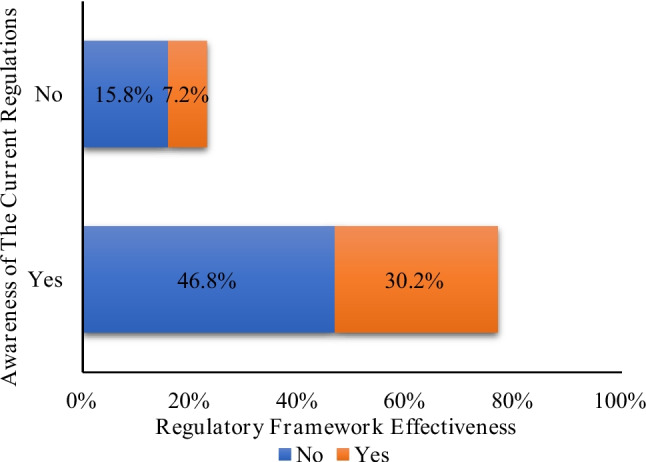
Awareness and effectiveness of the current regulations governing community pharmacies

The survey data presented in Fig. 3, provides insights into the awareness and regulatory framework effectiveness of the current regulations governing community pharmacies in Saudi Arabia. The results indicate that majority of participants (77%) were aware of these regulations. However, a notable discrepancy emerges when examining their opinions on the effectiveness of the current regulatory framework. While 46.8% of respondents believe the system is not effective, only 30.2% consider it effective. On the other hand, a smaller proportion (23%) were unaware of the existing regulations. Yet, even among this group, 15.8% still view the current framework as ineffective, compared to just 7.2% who find it effective.

When combining responses across groups, the data reveals that a substantial majority (62.6%) of all respondents perceive the current regulatory framework as ineffective (46.8% (aware & ineffective) + 15.8% (unaware & ineffective) = 62.6%), while only 37.4% view it as effective (30.2% and 7.2%).

This data highlights a knowledge gap in the dissemination of information related to community pharmacy regulations in Saudi Arabia. It, also, raises concerns of the effectiveness of the current regulatory framework.

For the fourth section of the survey, the data shows a clear preference for the Saudi Food and Drug Authority (SFDA) as the future regulator, with 107 respondents (77.0%) choosing SFDA, while only 32 respondents (23.0%) prefer the Ministry of Health. When asked whether SFDA would be better for regulating community pharmacies in the future, 112 participants (80.6%) answered"Yes"while 27 (19.4%) answered"No". This strong preference for SFDA suggests that significant respondents believe this organization is better equipped to handle regulatory responsibilities in the healthcare sector.

**Fig. 4:**
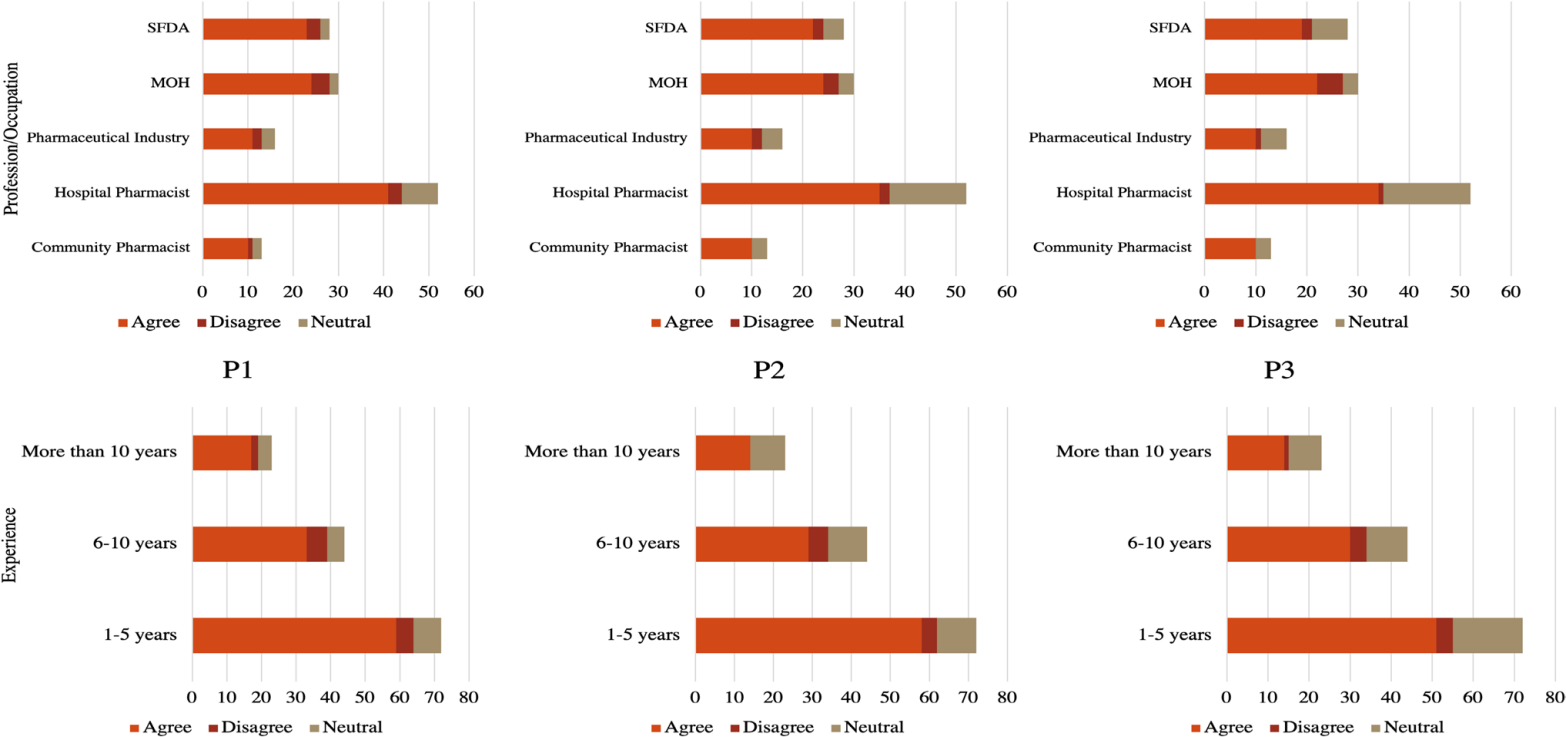
Perceptions toward inclusion of community pharmacies under SFDA regulation per profession/occupation and experience. P1, P2, and P3 are the questions where; P1: Enhancement of safety and quality standards for medications provided by community pharmacies under SFDA regulation, P2: Improvement in the oversight of pharmaceutical practices in community pharmacies under SFDA regulation, P3: Strengthening of public trust in community pharmacy services under SFDA regulation

The perceptions toward inclusion of community pharmacies under SFDA regulation per Profession/Occupation and Experience are found in Fig. 4. The questions are as follow; P1: Enhance the safety and quality standards for medications provided by community pharmacies, P2: Improve the oversight of pharmaceutical practices in community pharmacies, P3: Strengthen the public trust in community pharmacy services.

The significant agreement rates observed for key regulatory outcomes demonstrate broad professional consensus regarding SFDA's potential regulatory benefits. Specifically, 78.4% of respondents agreed on enhanced safety and quality standards for medications (P1), 72.7% agreed on improved oversight of pharmaceutical practices (P2), and 68.4% agreed on strengthened public trust in community pharmacy services (P3). These consistently high agreement levels across fundamental regulatory domains indicate widespread professional recognition that transitioning community pharmacy oversight to SFDA would yield measurable improvements in medication safety protocols, pharmaceutical practice standardization, and public confidence in community pharmacy services.

The positive response to improving adverse event reporting (P4: 79.1% agreement) demonstrates professional recognition of SFDA's potential role in enhancing pharmacy service quality and safety monitoring. Only 33.8% agreed that SFDA regulation would burden community pharmacies with compliance requirements, while 31.7% agreed stakeholder resistance, for P5 and P6, respectively.

### Statistical analysis

The statistical analysis reveals significant variations in regulatory perspectives across healthcare sectors, where (82.7%) across different professions indicate that MOH is currently regulating community Pharmacies. The significant association between profession and current regulator (p = 0.0067, x^2^ = 27.4177) suggests a clear relationship between professional roles and regulatory oversight perspectives. This Chi square value indicates a meaningful influence of professional background on regulatory views. Also, a significant Chi square value (p = 0.0006, x^2^ = 17.3465), shows 63% of respondents indicating that the current regulations do not resolve the community pharmacies challenges.

Despite the substantial majority of respondents indicating preference for the Saudi Food and Drug Authority (SFDA) as the future regulatory body (77.0%) and expressing that SFDA would be better for improvement of pharmaceutical care services (80.6%), no statistically significant associations were observed between these preferences and respondents'demographic characteristics. Chi-square analyses revealed non-significant relationships between future regulator preference and professional background (χ^2^ = 4.0388, p = 0.4008), as well as between the perception that SFDA would be better for improvement of pharmaceutical care services and profession (χ^2^ = 2.9052, p = 0.5738). Similarly, years of experience demonstrated no significant association with either future regulator preference (χ^2^ = 2.1639, p = 0.3389) or the view that SFDA would be better for improvement of pharmaceutical care services (χ^2^ = 1.75, p = 0.4169). These findings suggest that support for SFDA's regulatory role transcends professional boundaries and experience levels, indicating broad consensus across different stakeholder groups within the pharmaceutical sector.

The weak associations across professional groups for both P1 (Cramer's V = 0.1226, p = 0.8408), P2 (Cramer's V = 0.1668, p = 0.4594), and P3 (Cramer's V = 0.2069, p = 0.1555), suggest that support for these fundamental regulatory objectives transcends professional boundaries. Notably, community pharmacists showed particularly strong support (> 75% agreement) for all propositions. The consistent agreement rates across professional groups, as indicated by the low Cramer's V values, suggest widespread recognition of the potential benefits of standardized regulatory oversight for community pharmacies. Majority of respondents across both experiences and occupation with P1, P2, and P3 (78.4%, 72.7, and 68.4% respectively), agreed on the improvement SFDA regulation will produce for community pharmacies.

**Fig. 5:**
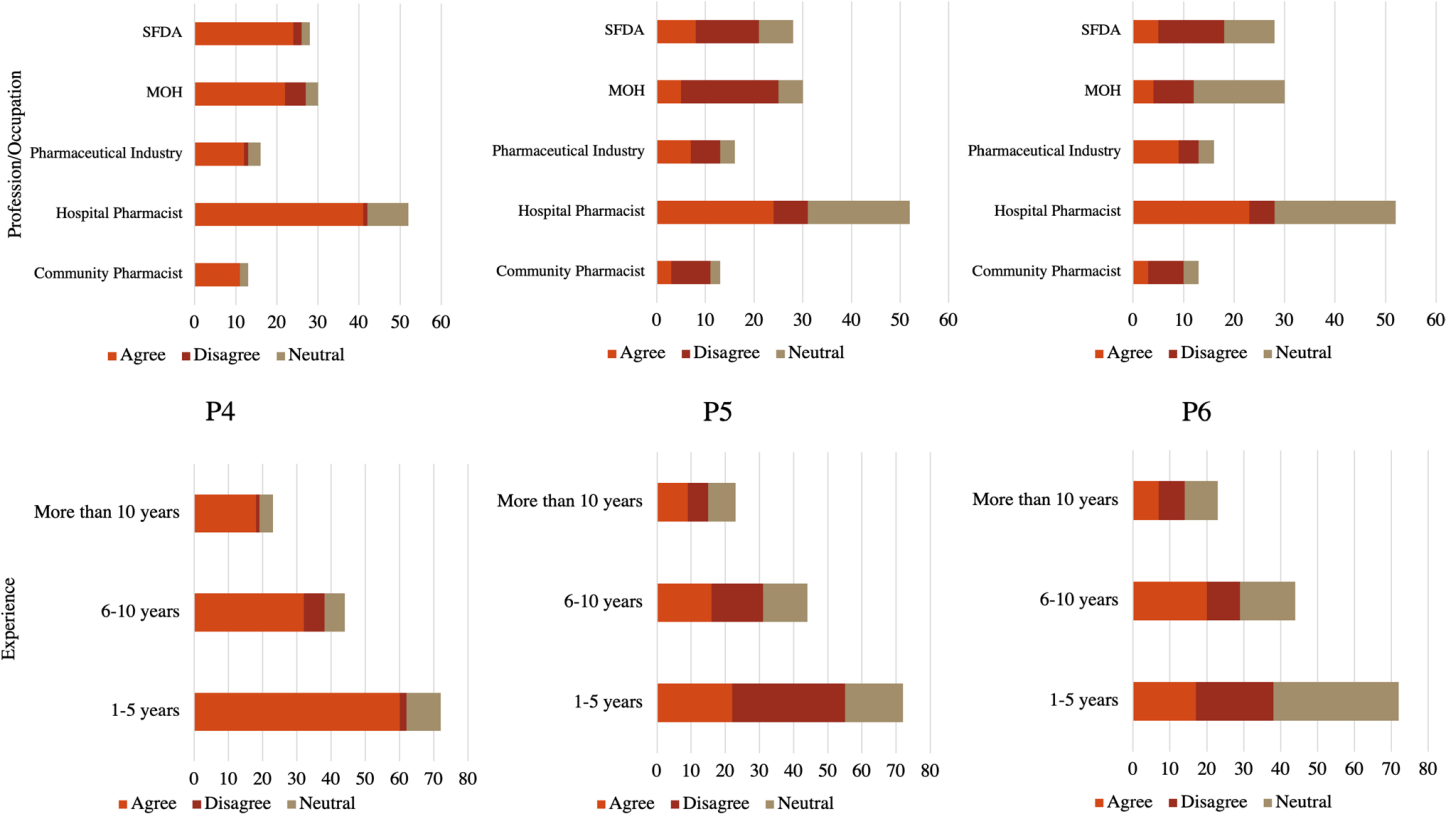
Perceptions toward inclusion of community pharmacies under SFDA regulation per profession/occupation and experience. P4, P5, and P6 are the questions where; P4: Efficient reporting and surveillance of adverse events or product recalls under SFDA regulation, P5: Production of a potential burden on community pharmacies in terms of compliance and documentation under SFDA regulation, P6: Induction of possible resistance or opposition from stakeholders within the pharmaceutical industry under SFDA regulation

The moderate association strength for P4 (Cramer's V = 0.1915, p = 0.2516) indicates some variation in professional perspectives regarding adverse event reporting systems. Experience levels showed similar patterns (83.3%, 72.7%, and 78.3% respectively) agreement for P4. The weak association between experience and P4 responses (Cramer's V = 0.1436, p = 0.2202) suggests that views on safety monitoring systems are relatively consistent across experience levels, Fig. 5. Strong associations were found across professional groups for both P5 (x^2^ = 28.4898, Cramer's V = 0.3201, p = 0.0004) and P6 (x^2^ = 30.2307, Cramer's V = 0.3298, p = 0.0002), indicating substantial differences in how various sectors view implementation challenges. These relatively high Chi Square and Cramer's V values suggest that professional background significantly influences perspectives on regulatory burden and resistance. Among Community pharmacists’ group, majority (61.5%, 53.9%) of respondents did not agree on question P5 and P6, respectively.

## Discussion

### Current regulatory framework: strengths and weaknesses

The assessment of Saudi Arabia's current regulatory framework for community pharmacies reveals both inherent strengths and significant limitations. Our findings indicate that while 77% of participants are aware of existing regulations, a substantial majority (62.6%) believe these regulations inadequately address the challenges faced by community pharmacies. This disconnects between awareness and effectiveness suggests that the issue lies not in the dissemination of regulatory information, but rather in the regulatory structure itself.

The current MOH oversight demonstrates strengths in terms of integrated healthcare policy implementation and established administrative processes. The high level of regulatory awareness among professionals indicates successful communication channels between the Ministry and stakeholders. However, the study reveals critical weaknesses in the existing framework. The perception that healthcare services provided by community pharmacies are subpar (62.6% of respondents) suggests that MOH oversight may be stretched thin across its numerous healthcare responsibilities.

When compared to similar healthcare systems in the MENA region, such as the UAE and Jordan, Saudi Arabia's single MOH oversight approach for community pharmacies appears increasingly outdated. The UAE's multi-tiered regulatory system, involving both federal and emirate-level authorities, demonstrates how specialized oversight can enhance pharmaceutical service quality. Similarly, Jordan's Food and Drug Administration model shows the benefits of dedicated pharmaceutical regulation (Main Ministry of Health Office 2019; Dubai Health Authority 2021; Main Ministry of Health 2015; Main Ministry of Health Office 2013).

The current framework's limitations are particularly evident in three areas: quality control, standardization of practices, and enforcement mechanisms. Participants highlighted challenges in maintaining consistent standards across different regions, suggesting that centralized MOH control may not provide sufficient localized oversight. Furthermore, the existing system appears to lack robust mechanisms for handling medication safety issues, adverse event reporting, and quality assurance protocols specific to community pharmacy operations.

### Implications of transition to SFDA regulation

The proposed transition to SFDA regulation represents a significant shift in Saudi Arabia's pharmaceutical governance landscape, with far-reaching implications for various stakeholders. Our findings reveal strong support for this change, with 77.% of participants preferring SFDA regulation and 80.6% believing it would improve healthcare services. This suggests a professional consensus regarding the potential benefits of specialized regulatory oversight.

The anticipated improvements under SFDA regulation are multifaceted. First, 78.4% of participants believe it would enhance medication safety and quality standards, likely due to SFDA's existing expertise in pharmaceutical product regulation. This specialized knowledge could lead to more targeted and effective oversight of community pharmacy operations. Second, 72.7% expect improved oversight of pharmaceutical practices, suggesting that SFDA's focused mandate could result in more efficient monitoring and enforcement mechanisms.

The transition could also address current systemic inefficiencies. SFDA's existing infrastructure for handling drug safety and quality issues could be extended to community pharmacy oversight, potentially creating a more integrated and responsive regulatory system. The authority's experience in implementing international standards and best practices could facilitate the modernization of community pharmacy services, aligning them with global benchmarks (Alhomaidan et al. 2024; Fouda et al. 2025).

However, the implications extend beyond operational improvements. The study indicates that 68.4%, 79.1% believe SFDA regulation would strengthen public trust in community pharmacy services and will lead to efficient reporting and surveillance of adverse effect, respectively. This increased confidence could lead to better utilization of pharmacy services, potentially reducing the burden on other healthcare facilities and improving overall healthcare accessibility.

The heterogeneous response patterns regarding compliance burden and stakeholder resistance merit careful consideration for implementation planning. Notably, 38.8% of respondents disagreed that SFDA regulation would impose additional compliance burdens on community pharmacies, while 26.6% disagreed that the transition would encounter stakeholder resistance. These findings suggest that a minority of participants perceive the regulatory transition as feasible without significant operational challenges or organizational opposition, though the divided responses indicate the need for strategic change management approaches to address varying stakeholder concerns.

The stakeholder Comments regarding the prospective inclusion of community pharmacies under (SFDA) regulation reveals diverse perspectives within the pharmaceutical sector. Particularly, an observation from a former community pharmacy practitioner highlighted the historical challenges in regulatory compliance, attributing these difficulties to inadequate management protocols. While some stakeholders expressed reservations about implementation feasibility, citing the sector's five-decade operational history as a potential barrier to systemic change, others recognized SFDA's potential to establish more structured organizational frameworks. Respondents made a notable distinction between product regulation, which was generally perceived as adequate, and practice regulation, which was identified as requiring enhancement. The stakeholder commentary acknowledged the substantial scope of such regulatory transition, suggesting the necessity for a longitudinal implementation strategy. This indicates the importance of developing a comprehensive, phased approach to regulatory reform that accounts for both immediate safety requirements and long-term operational sustainability.

### Statistical analysis

The research examined several key dimensions of regulatory oversight, with the efficient reporting system (P4) dimension emerging as the most positively perceived aspect, garnering 79.1% agreement (110 respondents). This strong endorsement of the reporting system suggests stakeholders find the SFDA's mechanisms for documentation and communication to be effective and user-friendly. Close behind was the safety and quality standards dimension, with 78.4% agreement (109 respondents), indicating strong confidence (P1) in SFDA's ability to maintain and enforce appropriate pharmaceutical standards. The general oversight improvement dimension (P2) also received substantial support at 72.7% (101 respondents), though notably lower than the other two dimensions.

The statistical analysis revealed interesting patterns in how different professional groups perceived the regulatory framework. The absence of significant associations in the chi-square tests between professional roles and perceptions of both efficient reporting and safety standards (p > 0.05) suggests a remarkable consistency in views across different professional categories. This uniformity of opinion across diverse stakeholder groups strengthens the credibility of the positive perceptions, as it indicates that support for SFDA regulation.

An intriguing aspect of the findings relates to the relationship between professional experience and support for SFDA regulation. The data reveals an interesting inverse relationship between years of experience and support levels. Professional experience and support for future SFDA regulation of community pharmacies, illustrated through declining support percentages across experience brackets: 81.9% for 1–5 years, 72.7% for 6–10 years, and 69.6% for those with over 10 years of experience. This pattern suggests several interpretations: professionals with 1–5 years of experience likely received education emphasizing regulatory compliance and standardized practices, while those with 6–10 years represent a transitional group balancing regulatory benefits with established practices. The lowest support among professionals with over 10 years of experience may reflect concerns about disrupting proven workflows rather than opposition to oversight itself. Notably, despite this inverse relationship, all experience groups maintain relatively high support levels (~ 70%), indicating broad acceptance of SFDA regulation across the professional spectrum. These findings suggest the need for targeted implementation approaches that leverage newer professionals'enthusiasm while addressing experienced practitioners'concerns through inclusive dialogue. The strong support among newer professionals is particularly significant as it suggests a positive outlook for the future of pharmacy regulation, as these professionals will increasingly occupy leadership positions in the field.

The study's findings have important implications for the transition of community pharmacy regulation to SFDA oversight. The weak associations in safety and quality measures (P1-P4) contrasted with strong associations in implementation concerns (P5-P6) indicate that while there's broad agreement on regulatory goals, there are significant differences in views about implementation challenges. The relationship between experience and the community pharmacies current services (Cramer's V = 0.2479, p = 0.0140) suggests that regulatory transition strategies should consider varying professional experience levels. The high awareness levels (77.0%) provide a strong foundation for stakeholder engagement, though the varying satisfaction levels with current regulation (37.4%) indicate the importance of addressing existing system weaknesses.

The variations in perceptions across professional roles and experience levels suggest that a one-size-fits-all approach to implementation may be suboptimal. Instead, a nuanced strategy that acknowledges and addresses the specific concerns of different stakeholder groups while building on the broad support for quality and safety improvements may be more effective. The statistical patterns observed suggest that successful implementation will require careful attention to operational concerns while leveraging the strong support for enhanced quality and safety standards.

### Future directions and recommendations

Based on our findings, several key recommendations emerge for the successful implementation of regulatory reform in Saudi Arabia's community pharmacy sector. First, a phased transition approach to SFDA regulation is advisable, allowing for systematic evaluation and adjustment of regulatory mechanisms. This could begin with pilot programs in one main city, followed by gradual expansion to other regions, enabling stakeholders to adapt to new requirements while maintaining service continuity.

Specific policy recommendations include developing a comprehensive regulatory framework that addresses the identified gaps in current oversight. This should encompass clear guidelines for quality assurance, standardized operating procedures, and robust mechanisms for monitoring compliance. The framework should also incorporate modern technological solutions for surveillance and reporting, building on SFDA's existing capabilities in pharmaceutical product tracking and safety monitoring.

Professional development and training programs should be established to support the transition. These programs should focus on familiarizing pharmacy professionals with new regulatory requirements, updating standard operating procedures, and enhancing quality management skills. Particular attention should be paid to smaller community pharmacies that may require additional support in meeting new regulatory standards.

Future research directions should include longitudinal studies to track the impact of regulatory changes on service quality and patient outcomes. Cost–benefit analyses of the transition would be valuable for policy refinement and resource allocation. Additionally, comparative studies with other countries that have undergone similar regulatory reforms could provide valuable insights for implementation strategies.

Stakeholder engagement should remain a priority, with regular feedback mechanisms established to identify and address implementation challenges. This could include the creation of advisory committees representing different sectors of the pharmacy profession, ensuring that regulatory development remains responsive to practical needs while maintaining high standards of safety and quality.

### Study limitations and methodological considerations

While this study provides valuable insights into stakeholders'perspectives on regulatory reform, several methodological limitations warrant careful consideration. The cross-sectional nature of our research design, while appropriate for capturing current attitudes, prevents us from understanding how perceptions might evolve over time or in response to incremental regulatory changes. This temporal limitation is particularly relevant given the dynamic nature of healthcare regulation and ongoing reforms in Saudi Arabia's healthcare sector.

Our sample composition presents both strengths and limitations. Although we achieved a diverse representation of pharmaceutical professionals, the predominance of hospital pharmacists (37.4%) and regulatory personnel may have introduced certain biases. The relatively lower representation of community pharmacists, who would be most directly affected by regulatory changes, could mean that some practical implementation challenges remain unexplored. Additionally, the young workforce profile, with 51.8% having 1–5 years of experience, might not fully capture the historical context and long-term perspectives on regulatory evolution. Furthermore, Cronbach's alpha coefficient (α = 0.66) that fell below the conventional threshold of 0.70, suggesting potential heterogeneity among questionnaire items, which may affect measurement precision. Additionally, while the sample size (n = 139) was sufficient for basic statistical analyses, it presents limitations regarding generalizability of the findings to the target population.

The reliance on self-reported data introduces potential social desirability bias, particularly regarding questions about current service quality and regulatory preferences. Participants might have been influenced by professional affiliations or institutional loyalties when expressing their views on regulatory oversight. Furthermore, the online distribution of the survey, while efficient, may have limited participation from professionals with limited digital access or technological proficiency.

The study's scope was also constrained by the limited availability of previous research on community pharmacy regulation in Saudi Arabia, making it difficult to establish trend comparisons or validate findings against historical data. The ambiguity in international guidance regulations posed challenges in benchmarking our findings against global standards and best practices.

## Conclusion

The study participants'overwhelming preference for SFDA regulation signifies a recognition among pharmacy professionals of the importance of specialized regulatory oversight. The study's diverse sample of participants, including hospital and community pharmacists, as well as regulatory professionals, lends credibility to the findings and ensures a comprehensive understanding of the regulatory needs of the sector. This inclusivity reflects the importance of gathering insights from various stakeholders to inform regulatory decision-making.

The identified areas of improvement within the current regulatory framework highlight specific challenges that community pharmacies face, such as operational issues and the quality of healthcare services. These findings highlight the need for regulatory reforms that address these concerns effectively and provide a strong foundation for the future of pharmaceutical regulation in Saudi Arabia.

The anticipated transition towards SFDA regulation aligns Saudi Arabia with global best practices in pharmaceutical regulation, ultimately enhancing public trust, accessibility to medications, and market integrity. By adopting international standards and tailoring them to the unique context of the country, Saudi Arabia can create a regulatory framework that promotes effective, transparent, and responsive pharmaceutical care.

By addressing the identified challenges and aligning with global best practices, Saudi Arabia can pave the way for an enhanced pharmaceutical sector that prioritizes safety, quality, and accessibility for all its citizens.

## Supplementary Information

Below is the link to the electronic supplementary material.

Supplementary file1 (DOCX 23 KB)

## Data Availability

The authors confirm that the data supporting the findings of this study are available within the article and its supplementary materials.
