# Supplementary material for: Assessment of the feasibility of including community pharmacies under the regulation of the saudi food and drug authority
Source: Saudi Pharm J. 2025 Oct 2;33(5):36. doi: 10.1007/s44446-025-00036-0 (PMC12491131; doi:10.1007/s44446-025-00036-0)
Supplement: Supplementary file 1 — Supplementary file1 (DOCX 23 KB) [file 44446_2025_36_MOESM1_ESM.docx]

**Structured Results Table for the knowledge of current regulator**

| **Current_regulator** | **Count of Profession/Occupation** |
| --- | --- |
| **Ministry of Health** | 115 |
| **SFDA** | 17 |
| **Others** | 4 |
| **I do not know** | 3 |
| **Grand Total** | **139** |

**Structured Results Table for future regulator and Healthcare services improvement under SFDA**

| **Regulating community pharmacies under the Saudi Food and Drug Authority would improve healthcare services in Saudi Arabia?** | **Saudi Food and Drug Authority** | **Ministry of Health** | **Grand Total** |
| --- | --- | --- | --- |
| **Yes** | 105 | 7 | 112 |
| **No** | 2 | 25 | 27 |
| **Grand Total** | **107** | **32** | **139** |

**Table 1: Summary of Perception Outcomes Regarding SFDA Regulation of Community Pharmacies**

| **Perception Code** | **Question Description** | **Agree n (%)** | **Neutral n (%)** | **Disagree n (%)** | **Total n** | **Cramer's V** | **p-value** | **Statistical Significance** |
| --- | --- | --- | --- | --- | --- | --- | --- | --- |
| **P1** | Enhancement of safety and quality standards for medications provided by community pharmacies under SFDA regulation | 109 (78.4) | 17 (12.2) | 13 (9.4) | 139 | 0.1226 | 0.8408 | Not significant |
| **P2** | Improvement in the oversight of pharmaceutical practices in community pharmacies under SFDA regulation | 101 (72.7) | 29 (20.9) | 9 (6.5) | 139 | 0.1668 | 0.4594 | Not significant |
| **P3** | Strengthening of public trust in community pharmacy services under SFDA regulation | 95 (68.3) | 35 (25.2) | 9 (6.5) | 139 | 0.2069 | 0.1555 | Not significant |
| **P4** | Efficient reporting and surveillance of adverse events or product recalls under SFDA regulation | 110 (79.1) | 20 (14.4) | 9 (6.5) | 139 | 0.1915 | 0.2516 | Not significant |
| **P5** | Production of a potential burden on community pharmacies in terms of compliance and documentation under SFDA regulation | 47 (33.8) | 38 (27.3) | 54 (38.8) | 139 | 0.3201 | 0.0004 | **Significant** |
| **P6** | Induction of possible resistance or opposition from stakeholders within the pharmaceutical industry under SFDA regulation | 44 (31.7) | 58 (41.7) | 37 (26.6) | 139 | 0.3298 | 0.0002 | **Significant** |

**Table 2: Perception Outcomes by Professional Category**

**P1: Enhancement of Safety and Quality Standards**

| **Professional Category** | **Agree n (%)** | **Neutral n (%)** | **Disagree n (%)** | **Total n** |
| --- | --- | --- | --- | --- |
| Hospital Pharmacists | 41 (78.8) | 8 (15.4) | 3 (5.8) | 52 |
| MOH Regulatory | 24 (80.0) | 2 (6.7) | 4 (13.3) | 30 |
| SFDA Regulatory | 23 (82.1) | 2 (7.1) | 3 (10.7) | 28 |
| Pharma Company | 11 (68.8) | 3 (18.8) | 2 (12.5) | 16 |
| Community Pharmacists | 10 (76.9) | 2 (15.4) | 1 (7.7) | 13 |

**P2: Improvement in Oversight**

| **Professional Category** | **Agree n (%)** | **Neutral n (%)** | **Disagree n (%)** | **Total n** |
| --- | --- | --- | --- | --- |
| Hospital Pharmacists | 35 (67.3) | 15 (28.8) | 2 (3.8) | 52 |
| MOH Regulatory | 24 (80.0) | 3 (10.0) | 3 (10.0) | 30 |
| SFDA Regulatory | 22 (78.6) | 4 (14.3) | 2 (7.1) | 28 |
| Pharma Company | 10 (62.5) | 4 (25.0) | 2 (12.5) | 16 |
| Community Pharmacists | 10 (76.9) | 3 (23.1) | 0 (0.0) | 13 |

**P3: Strengthening Public Trust**

| **Professional Category** | **Agree n (%)** | **Neutral n (%)** | **Disagree n (%)** | **Total n** |
| --- | --- | --- | --- | --- |
| Hospital Pharmacists | 34 (65.4) | 17 (32.7) | 1 (1.9) | 52 |
| MOH Regulatory | 22 (73.3) | 3 (10.0) | 5 (16.7) | 30 |
| SFDA Regulatory | 19 (67.9) | 7 (25.0) | 2 (7.1) | 28 |
| Pharma Company | 10 (62.5) | 5 (31.3) | 1 (6.3) | 16 |
| Community Pharmacists | 10 (76.9) | 3 (23.1) | 0 (0.0) | 13 |

**P4: Efficient Reporting and Surveillance**

| **Professional Category** | **Agree n (%)** | **Neutral n (%)** | **Disagree n (%)** | **Total n** |
| --- | --- | --- | --- | --- |
| Hospital Pharmacists | 41 (78.8) | 10 (19.2) | 1 (1.9) | 52 |
| MOH Regulatory | 22 (73.3) | 3 (10.0) | 5 (16.7) | 30 |
| SFDA Regulatory | 24 (85.7) | 2 (7.1) | 2 (7.1) | 28 |
| Pharma Company | 12 (75.0) | 3 (18.8) | 1 (6.3) | 16 |
| Community Pharmacists | 11 (84.6) | 2 (15.4) | 0 (0.0) | 13 |

**P5: Potential Compliance Burden**

| **Professional Category** | **Agree n (%)** | **Neutral n (%)** | **Disagree n (%)** | **Total n** |
| --- | --- | --- | --- | --- |
| Hospital Pharmacists | 24 (46.2) | 21 (40.4) | 7 (13.5) | 52 |
| MOH Regulatory | 5 (16.7) | 5 (16.7) | 20 (66.7) | 30 |
| SFDA Regulatory | 8 (28.6) | 7 (25.0) | 13 (46.4) | 28 |
| Pharma Company | 7 (43.8) | 3 (18.8) | 6 (37.5) | 16 |
| Community Pharmacists | 3 (23.1) | 2 (15.4) | 8 (61.5) | 13 |

**P6: Stakeholder Resistance**

| **Professional Category** | **Agree n (%)** | **Neutral n (%)** | **Disagree n (%)** | **Total n** |
| --- | --- | --- | --- | --- |
| Hospital Pharmacists | 23 (44.2) | 24 (46.2) | 5 (9.6) | 52 |
| MOH Regulatory | 4 (13.3) | 18 (60.0) | 8 (26.7) | 30 |
| SFDA Regulatory | 5 (17.9) | 10 (35.7) | 13 (46.4) | 28 |
| Pharma Company | 9 (56.3) | 3 (18.8) | 4 (25.0) | 16 |
| Community Pharmacists | 3 (23.1) | 3 (23.1) | 7 (53.8) | 13 |

**Table 3: Perception Outcomes by Years of Experience**

**Overall Agreement by Experience Level**

| **Experience Level** | **P1 Agree (%)** | **P2 Agree (%)** | **P3 Agree (%)** | **P4 Agree (%)** | **P5 Agree (%)** | **P6 Agree (%)** | **Total n** |
| --- | --- | --- | --- | --- | --- | --- | --- |
| 1-5 years | 59 (81.9) | 58 (80.6) | 51 (70.8) | 60 (83.3) | 22 (30.6) | 17 (23.6) | 72 |
| 6-10 years | 33 (75.0) | 29 (65.9) | 30 (68.2) | 32 (72.7) | 16 (36.4) | 20 (45.5) | 44 |
| >10 years | 17 (73.9) | 14 (60.9) | 14 (60.9) | 18 (78.3) | 9 (39.1) | 7 (30.4) | 23 |
